# EEG analysis and mobile robot as tools for emotion characterization in autism

**DOI:** 10.1186/1753-6561-8-S4-P85

**Published:** 2014-10-01

**Authors:** Christiane Goulart, Javier Castillo, Carlos Valadão, Teodiano Bastos, Eliete Caldeira

**Affiliations:** 1 Federal University of Espirito Santo, Vitória, Brazil

## Background

Autism or "Autism Spectrum Disorder" is characterized by manifestations of impairments in social behavior, stereotyped movements, difficulty in communication and interaction with people. This paper presents a system composed of a mobile robot to generate interactive tasks with autistic children, and an EEG (electroencephalography) equipment to get brain information to characterize their emotions.

## Methods

The mobile robot is equipped with a laser sensor to detect and locate the position of the child, in order to obtain distances to her/him, and a computer for brain signal processing and to schedule rules for the interaction. In addition to that, an wireless EEG cap is placed over the child's head in order to capture the child's brain signals during the interaction process, through electrodes specifically placed on the frontal cortex at positions Fp1, Fp2, F3 and F4, according to the international system 10-20, one of the most widely used electrode placement system on the skull. The robotic implementation allows two modes of interaction, depending on the interaction level with the child. Through the "dog" mode and "follower" mode, the robot identifies the position of the child and moves to where he/she is, keeping a safe distance of interaction. When the child has little interest in what happens around him/her, the robot ("dog" mode) approaches and moves away from him/her, attracting his/her attention. When the child shows some interest in interacting with the robot, this ("follower" mode) follows her/him if she/he moves away from it. On the other hand, if the child approaches the robot, this will move away, keeping the distance of interaction, and stops in order to start the interaction. During the whole process, the child's brain signals are captured by a wireless EEG cap and analyzed by the computer. The methods to evaluate autistic child during the interaction with the robot involve the Goal Attainment Scale (GAS) and a behavioral evaluation, which takes into account the child's emotional state.

## Results

The system of detection and location of the child is effective to obtain his/her coordinates. Thus, a safe distance is defined in order to preserve the physical integrity of the child during her/his interaction with the robot. Often the difficulty in communication and interaction with people prevents knowledge about feelings and emotions that individuals with autism have. The use of EEG allows monitoring the child's brain signals and making a characterization of emotions linked to aspects of the interaction with robots.

## Conclusions

The implementation of the two robotic modes of interaction ("dog" mode and "follower" mode) assists directly the process of social evolution of autistic children, as a teaching tool for parents, teachers, carers, therapists and researchers. By evaluating the detection of autistic child's mental states by EEG along with a behavioral assessment, it is possible to establish the corresponding emotions and analyze the evolution of the interaction between the child and the robot efficiently.
